# The impact of moxibustion on brain function in vascular dementia: a neuroimaging study involving amplitude of low-frequency fluctuations and functional connectivity

**DOI:** 10.3389/fneur.2025.1676496

**Published:** 2025-12-18

**Authors:** Yating Zhang, Zhilin Huang, Ping Wang, Sicheng Liu, Yinqiu Fan, Bixiang Zha, Xiaodi Qiao, Jun Yang

**Affiliations:** 1 The First Clinical Medical Collage of Anhui University of Chinese Medicine, Hefei, China; 2 The First Affiliated Hospital of Anhui University of Chinese Medicine, Hefei, China

**Keywords:** acupuncture, vascular dementia, moxibustion, amplitude of low-frequency fluctuation, functional connectivity

## Abstract

**Object:**

On the basis of determining the clinical effect of moxibustion on vascular dementia (VD), this study explored the central mechanism of moxibustion on VD through fMRI.

**Method:**

Fifty-five VD patients formed the observation group (treated with moxibustion at GV20, GV14, and GV24; 20 min/acupoint, once every other day, three times a week) and 50 healthy individuals served as the control group. The Montreal Cognitive Assessment (MoCA) and Mini-Mental State Examination (MMSE) were administered, and fMRI data collected, pre- and post-treatment for the observation group, and once for the control group. Amplitude of low-frequency fluctuation (ALFF) was analyzed for pre-treatment fMRI data of both groups. Regions of interest (ROI) were defined by discrepant brain regions, and functional connectivity (FC) was analyzed for the observation group.

**Result:**

After moxibustion treatment, MoCA scores increased with a mean difference (MD) of 2.0385 (95% CI, 1.6867 to 2.3902), and MMSE scores with an MD of 3.3077 (95% CI, 2.7997 to 3.8156), both significantly (*p* < 0.01). Regarding fMRI findings, baseline ALFF alterations in VD patients vs. controls included increased values in the bilateral hippocampus (HIP), precuneus, and left middle temporal gyrus (MTG), and decreased values in the left posterior cingulate cortex (PCC) and insula (INS) (FDR-corrected *p* < 0.05). After moxibustion treatment, significant FC increases were observed: left PCC with right superior/inferior temporal gyrus, left INS with right superior temporal gyrus/superior occipital gyrus and left inferior temporal gyrus, left HIP with right superior temporal gyrus and left PCC, and right HIP with bilateral postcentral gyrus and left fusiform gyrus (GRF-corrected: voxel *p* < 0.005, cluster *p* < 0.05). Additionally, pre-treatment left MTG ALFF was negatively correlated with MMSE (*p* < 0.05, *r* = −0.486). Post-treatment, FC of left PCC-right superior/inferior temporal gyrus (*r* = 0.650/0.638), left HIP-left PCC (*r* = 0.456), and right HIP-left fusiform gyrus (*r* = 0.411) were positively correlated with MMSE (*p* < 0.05).

**Discussion:**

Moxibustion can effectively improve the cognitive dysfunction in VD patients, possibly by promoting DMN functional recovery and facilitating functional reorganization.

## 1 Introduction

Vascular dementia (VD) is a syndrome in which neurocognitive deficits arising from hypoperfusion of brain regions and other insidious vascular brain injury diseases are the main manifestations, and it is a chronic progressive disease (1). The prevalence of dementia among people aged 60 years and older in China is 6.0%, of which VD is 1.6%, and the prevalence is a common type of dementia second only to Alzheimer’s disease (AD) (2, 3). The main clinical symptoms of VD are impaired memory, executive ability, and language function, accompanied by significant thinking, calculation, emotional and behavioral disorders, which are characterized by a long duration of the disease, high disease burden, and high rate of disability, and there is no proven efficacious medication to block or slow down the progression of VD (4). Previous studies (5) have shown that early intervention can stop further progression of VD. Moxibustion, as a distinctive therapeutic modality in Traditional Chinese Medicine (TCM), boasts a history spanning over 2,500 years and has been extensively utilized in the management of various neurodegenerative disorders (6). Our previous research demonstrated that moxibustion therapy exhibits significant efficacy in treating VD, effectively improving cognitive function while promoting remyelination and mitigating neuronal apoptosis (7–11).

In recent years, neuroplasticity has been identified as one of the pivotal therapeutic targets for VD (12). Neuroplasticity refers to the ability of the nervous system to adapt to internal and external environmental changes by reorganizing its structure, function, and connections, and is a key repair mechanism for VD (13). The advancement of neuroimaging techniques has provided novel insights into the comprehension of this reparative mechanism. Functional magnetic resonance imaging (fMRI) technology, with the advantages of noninvasive real-time imaging and high resolution, has been used as an important means of studying the functional activities of the human brain and its dysfunctions, and it has also been widely utilized in the study of the central mechanism of action of acupuncture and moxibustion (14). The commonly used analysis methods of fMRI mainly include functional separation and functional integration, among which the functional separation algorithms include amplitude of low-frequency fluctuations (ALFF) and regional homogeneity (ReHo), which can explore the characteristics of neural function changes in the range of localized brain regions and functional connectivity (FC) analysis is one of the functional integration algorithms (15). A targeted whole-brain FC analysis can be performed based on the results of ALFF and ReHo. The organic combination of functional separation and functional integration enables more systematic brain function research. The organic combination of functional separation and functional integration enables brain function research more systematically. In this study, on the basis of observing the clinical efficacy of moxibustion in the treatment of VD, we analyzed the different brain regions between VD patients and healthy control using ALFF, and regarded the different brain regions as regions of interest (ROI), and observed the effects of moxibustion on the function of the brain network of VD patients by using the FC analytical method, with the purpose of revealing the partial central action mechanism of moxibustion in the treatment of VD patients.

## 2 Methods and analysis

### 2.1 Subjects design

The study was conducted at the Department of Acupuncture and Rehabilitation, Department of Neurology, the First Affiliated Hospital of Anhui University of Chinese Medicine, with recruitment period from January 2024 to January 2025. A total of 55 patients with VD were initially recruited, and 50 healthy volunteers were publicly recruited as the control group, matched for age, gender, and years of education with VD patients.

#### 2.1.1 Sample size

According to previous clinically relevant fMRI study (16), with high sample homogeneity, a single-group sample size of 12 cases can be statistically valid, and after further evaluation of the 55 patients included in the observation group (e.g., adherence issues, ability to adhere to the treatment, etc.), 30 patients with VD were ultimately included in this study as the moxibustion treatment group for clinical observation.

#### 2.1.2 Ethics approval

The protocol was approved by the Ethics Committee of the First Affiliated Hospital of Anhui University of Chinese Medicine (Ethics No. 2023AH-67) and registered with the China Clinical Trial Registry (ChiCTR2400079428).

#### 2.1.3 Diagnostic criteria

The diagnostic criteria for VD patients were developed with reference to the 2019 Chinese Guidelines for the Diagnosis and Treatment of Vascular Cognitive Impairment (17).

#### 2.1.4 Inclusion criteria

The detailed inclusion criteria were summarized as follows. Participants ① met the diagnostic criteria for VD; ② aged between 50 and 75 years, right-handed; ③ Montreal cognitive assessment (MoCA) scores of 17 or less; ④ conscious, without language impairment, and able to cooperate in completing the relevant scale; ⑤ had received no acupuncture and moxibustion treatment in the last 3 months; ⑥ voluntarily participated in this trial and signed the informed consent.

#### 2.1.5 Exclusion criteria

The exclusion criteria were those who ① had intracranial lesions other than cerebrovascular disease; ② had cognitive dysfunction caused by other non-vascular factors such as systemic diseases; ③ had a combination of serious other systemic diseases and depression in the elderly, which the investigator considered unsuitable for participation in the study; ④ were currently receiving other treatments that might affect the assessment of the efficacy of the treatment; ⑤ had MRI contraindications (pacemakers, stents, claustrophobia, etc.); ⑥ Inability to cooperate with the trial (e.g., inability to adhere to the entire trial, etc.).

### 2.2 Moxibustion treatment

**Figure 1:**
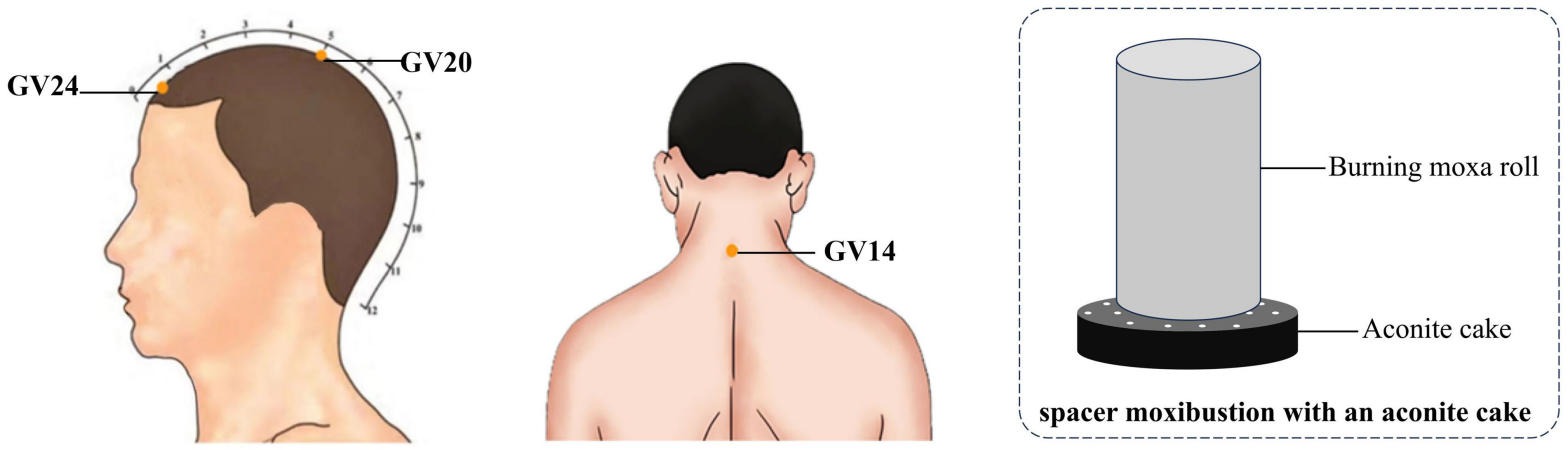
Locations of selected acupoints and schematic diagram of moxibustion treatment. The acupoints were including GV14 (*Dazhui*), GV20 (*Baihui*) and GV24 (*Shenting*).

The patients in the observation group were treated with the method of moxibustion, which was carried out by subject members who had been uniformly trained in standard operating procedures. Three acupoints, GV20 (Baihui), GV14 (Dazhui), and GV24 (Shenting), were selected for treatment, and the localization of the acupoints was referred to the national standard of the People’s Republic of China: Name and Location of Meridian Points (18). Operation method: the patient took a sitting position, using the moxa roll and aconite cake for moxibustion, in which the diameter of moxa roll was 1.8 cm long and 20 cm weighted about 24 g, and the diameter of aconite cake was 3 cm and 1 cm thick. GV20 uses spacer moxibustion with an aconite cake, place a burnt moxa roll on the aconite cake and then place the aconite cake on the acupoint for pressing moxibustion, at the same time, the preparation of the heat-resistant wide-mouthed bottle was placed in the proximity of the operation, and the ash was quickly flicked about every 2 min. GV14 and GV24 use hanging moxibustion, which involve placing moxa roll vertically above the acupoints for moxibustion and quickly flicking the ash about every 5 min. The temperature of the body surface was measured using a thermometer so that the moxibustion temperature was controlled at 45 °C ± 1. The treatment of moxibustion was 20 min, and the treatment was performed three times a week (Tuesday, Thursday and Saturday), 10 times for each course of treatment, and the observation group received a total of 3 courses of treatment. The locations of the selected acupoints and schematic diagram of the moxibustion treatment are shown in Figure 1.

### 2.3 Clinical efficacy measurement

In this study, the treatment effect was assessed by evaluating the degree of cognitive improvement using the Montreal Cognitive Assessment (MoCA) scale (19) and the Mini-mental State Examination (MMSE) scale (20), which were assessed in the observation group before and after treatment assessments. MoCA scale has a total score of 30 points, with less than 12 years of education adding one point to the total, with lower scores representing greater cognitive dysfunction. MMSE scale, based on the years of education criteria, assesses cognitive impairment as less than 17 points for illiteracy, less than 20 points for primary school, less than 22 points for secondary school, and less than 23 points for university.

### 2.4 Image data acquisition

The observation group took MRI data before and after treatment, and the healthy control group performed one MRI data acquisition after inclusion. All subjects’ MRI data were acquired using GE’s 8-channel skull coil 3.0 T scanner (Discovery MR750, United States) at the Imaging Centre of the First Affiliated Hospital of Anhui University of Chinese Medicine. The subjects had all metal and magnetic objects were removed from their bodies before the scan and were prepared with noise-canceling earplugs and sponge pads to hold their heads in place, and were instructed to keep their eyes closed and their whole body still during the scanning process. A routine scan was performed first and the collection continued after the results suggested that there were no abnormal changes in the brain such as hemorrhages or tumors.

Structure images were obtained using 3D T1 BRAVO sequence: repetition time (TR) = 8.2 ms, echo time (TE) = 3.2 ms, inversion time = 450 ms, turn angle (FA) = 12°, field of view (FOV) = 256 mm × 256 mm, matrix = 256 × 256, layer thickness = 1.0 mm, voxel = 1 mm × 1 mm × 1 mm, number of layers = 166 layers, total duration = 5 min30s. Gradient echo plane echo imaging sequence was used to collect resting-state fMRI: TR = 2,000 ms, TE = 35 ms, FA = 90°, FOV = 240 mm × 240 mm, matrix = 64 × 64, layer thickness = 3.0 mm, voxel = 3 mm × 3 mm × 3 mm, number of layers = 36 layers, 185 time points in total, total duration = 6 min 10 s.

### 2.5 Data processing and statistical analysis

Clinical data analysis: SPSS 26.0 software was used for statistical processing. Two-sample t test and Chi-square test were used to compare the matching degree of general information (age, gender, and years of education) of the subjects. Normally distributed continuous variables were analyzed using paired *t*-test, while non-normal continuous variables were analyzed using Wilcoxon rank-sum test. Differences were considered statistically significant at *p* < 0.05.

Preprocessing of fMRI data: the DPABI software (21) was used for pre-processing. Firstly, the raw data was changed from DICOM format to NIFTI format, and the data was checked one by one for artifacts or large head movements. Then the following processing was performed, ① removing the first 10 times; ② correcting slice timing; ③ excluding data with head movements of more than 3 mm or 3°; ④ normalizing to MNI template, voxel resampling to 3 mm × 3 mm × 3 mm resolution; ⑤ regressing interfering factors, including signals from head movements, cerebral white matter, and cerebrospinal fluid; ⑥ spatially smoothing with a 6-mm half-height, full-width Gaussian kernel; ⑦ keeping the 0.01 ~ 0.08 Hz low-frequency band signals.

The ALFF value calculation and analysis: smoothed preprocessed data were obtained via DPABI software for ALFF value calculation. For statistical analysis, two independent-samples t-tests were performed on the data of the observation group (before treatment) and the healthy control group using both DPABI and SPM12. The final results were corrected using the False Discovery Rate (FDR) method, with a significance threshold set at *p* < 0.05.

The FC value calculation and analysis: the differentiated brain regions obtained after ALFF analysis were used as ROIs, and the FC between ROIs and the whole brain was calculated using the DPABI software, and a paired-samples *t*-test was performed on the FC data between post-treatment and pre-treatment of the patients in the observation group. The final results were corrected by Gaussian random field (GRF) with voxel level *p* < 0.005 and cluster level *p* < 0.05.

Correlation analysis: Pearson’s correlation analysis was performed between pre-treatment ALFF and post-treatment FC values with MoCA and MMSE scores in the observation group.

## 3 Results

### 3.1 Demographics and MoCA, MMSE data

A total of 55 patients with VD and 50 healthy controls were recruited for this study. The demographic characteristics, including gender, age, and years of education, showed no statistically significant differences between VD patients and healthy controls (all *p* > 0.05). However, VD patients had significantly lower baseline MoCA scores compared to healthy controls (VD: 11.84 ± 1.46 vs. Healthy control: 28.14 ± 1.07, *p* < 0.001) (Table 1).

**Table 1:** Demographic characteristics of the study participants.

Characteristics of the participants	Healthy control (*n* = 50)	VD patients (*n* = 55)	*P*-value
Male, *n* (%)	21 (42%)	24 (43.6%)	0.866
Age, mean (SD), years	60.76 ± 6.25	62.42 ± 6.11	0.172
Education, mean (SD), years	9.26 ± 2.72	8.95 ± 2.89	0.572
MoCA scores, mean (SD)	28.14 ± 1.07	11.84 ± 1.46	<0.001

**Figure 2:**
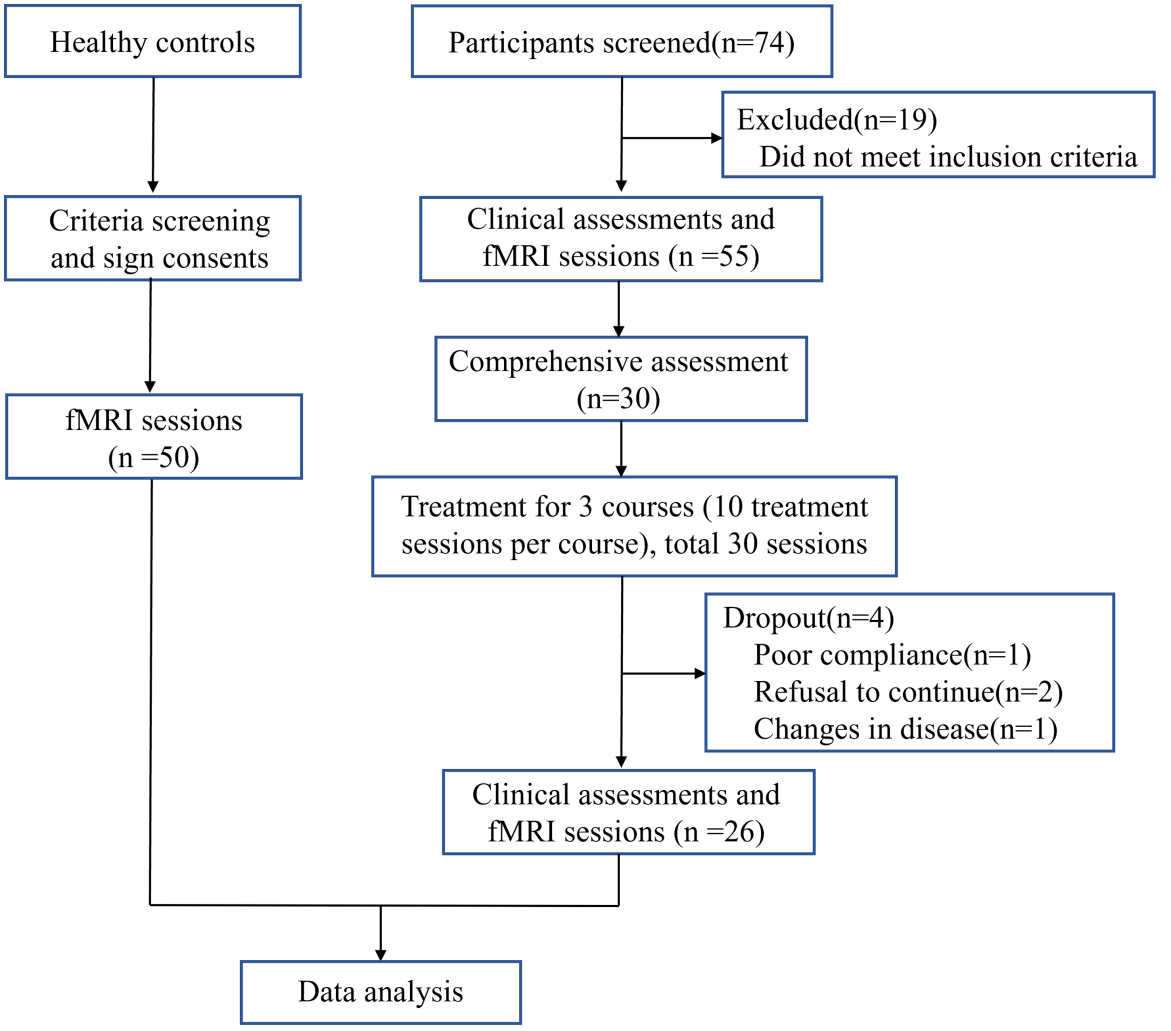
Screening Flowchart.

**Figure 3:**
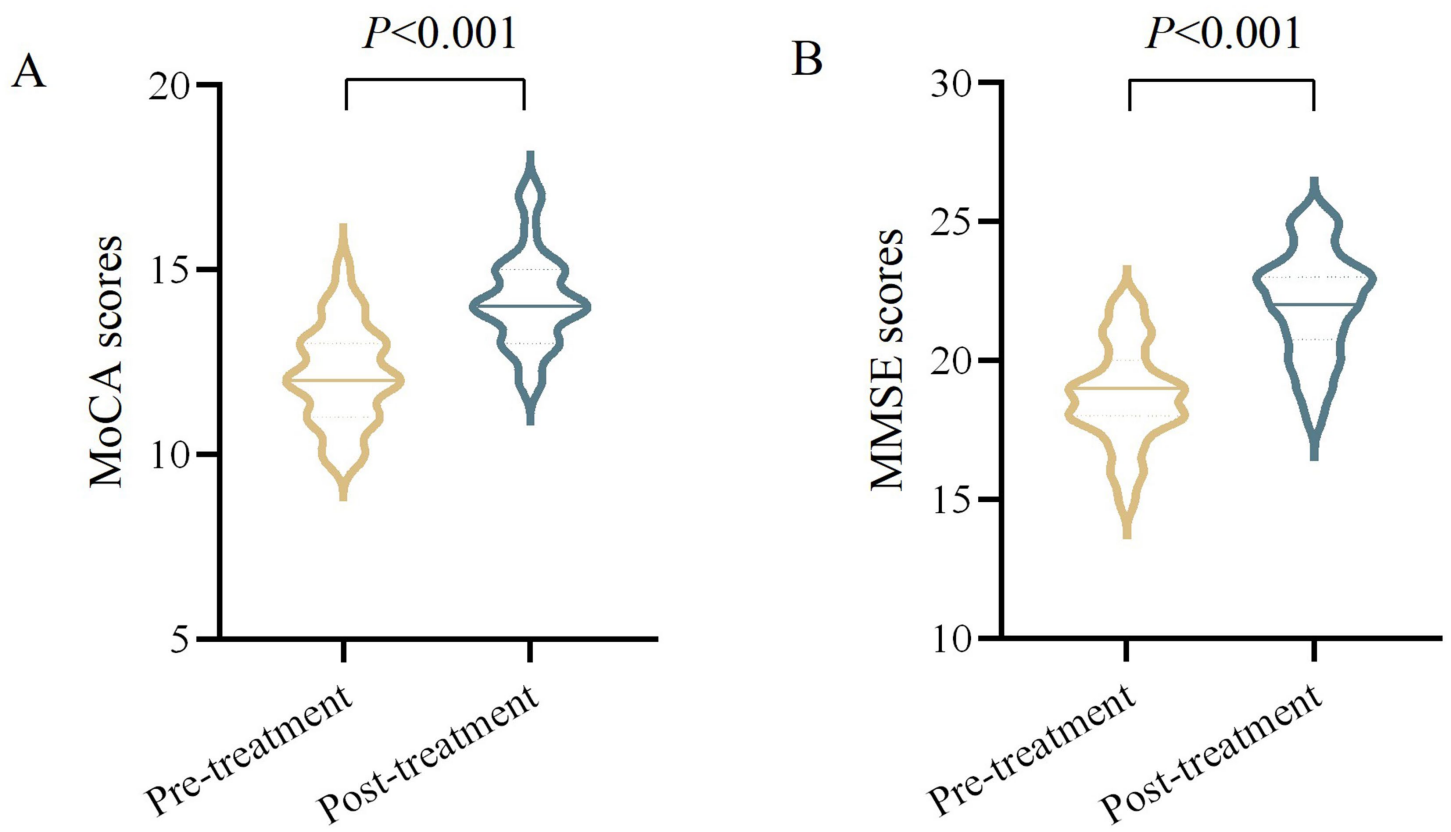
Comparison of MoCA and MMSE scores in VD patients before and after moxibustion treatment. **(A)** MoCA scores (pre-treatment vs. post-treatment, *p* < 0.001). **(B)** MMSE scores (pre-treatment vs. post-treatment, *p* < 0.001).

Included 30 cases of VD patients for treatment, 1 case was dropped due to poor compliance, 2 cases were dropped due to personal reasons for inability to adhere to the treatment, 1 case was discontinued due to the emergence of comorbidities that were assessed to be inappropriate to continue this study. The comprehensive screening procedure is delineated in Figure 2. Finally, 26 cases were included in the statistical analysis. After moxibustion treatment, the observation group demonstrated significant improvements in both MoCA and MMSE scores. Specifically, the mean difference (MD) in MoCA scores was 2.04 (95% confidence interval [CI]: 1.69 to 2.39, *p* < 0.001), and the MD in MMSE scores was 3.31 (95% CI, 2.80 to 3.82, *p* < 0.001) (Table 2 and Figure 3).

**Table 2:** Comparison of MoCA and MMSE scores of observation group before and after treatment.

Outcome measures	Mean (95% CI)	Mean difference (95% CI)	*P*-value
MoCA			
Pre-treatment	12.15 (11.62, 12.69)	NA	NA
Post-treatment	14.19 (13.68, 14.70)	2.04 (1.69, 2.39)	< 0.001
MMSE			
Pre-treatment	18.73 (18.01, 19.45)	NA	NA
Post-treatment	22.04 (21.27, 22.81)	3.31 (2.80, 3.82)	< 0.001

NA, not applicable.

### 3.2 ALFF contrasts

**Figure 4:**
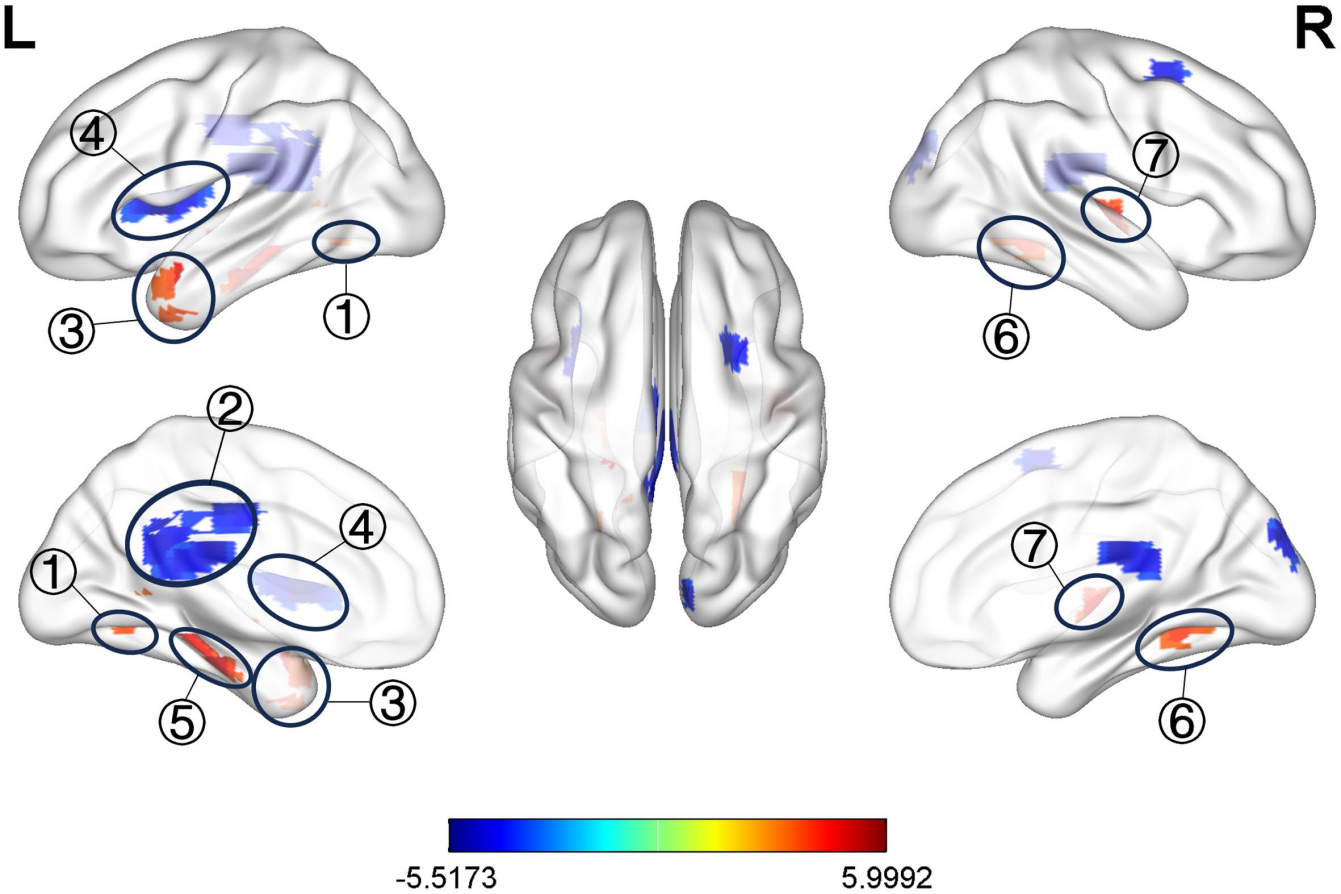
Significant difference in ALFF between observation group VD patients’ pre-treatment and healthy control. ① L. PCUN, ② L. PCC, ③ L. MTG, ④ L. INS, ⑤ L. HIP, ⑥ R. PCUN, ⑦ R. HIP.

Compared with the healthy control group, ALFF values of bilateral hippocampus (HIP), bilateral precuneus (PCUN), and left middle temporal gyrus (MTG) increased in the observation group before treatment, and ALFF values were decreased in the left posterior cingulate cortex (PCC), and left insula (INS) (Table 3 and Figure 4, *p* < 0.05, correction with FDR).

**Table 3:** Comparison of ALFF between observational group’s pre-treatment and healthy control.

Regions	Side	Voxels	MNI			*P*-value	*P*-value (peak intensity)
			*x*	*y*	*z*		
PCUN	L	87	−30	−51	12	0.006	4.909
PCC	L	74	−3	−30	21	0.007	−3.471
MTG	L	46	−45	0	−24	0.001	5.999
INS	L	37	−36	−3	9	0.045	−3.477
HIP	L	36	−21	−27	−24	0.001	5.661
PCUN	R	33	33	−39	3	0.024	4.254
HIP	R	32	45	−15	−3	0.002	5.144

The result was corrected for FDR with *P* < 0.05. MNI, Montreal Neurological Institute. A positive *T*-value indicates an increased ALFF, A negative *T*-value indicates a decreased ALFF.

### 3.3 FC contrasts

As ROIs, the discrepant brain regions obtained after ALFF analysis were used for whole-brain FC analysis.

**Figure 5:**
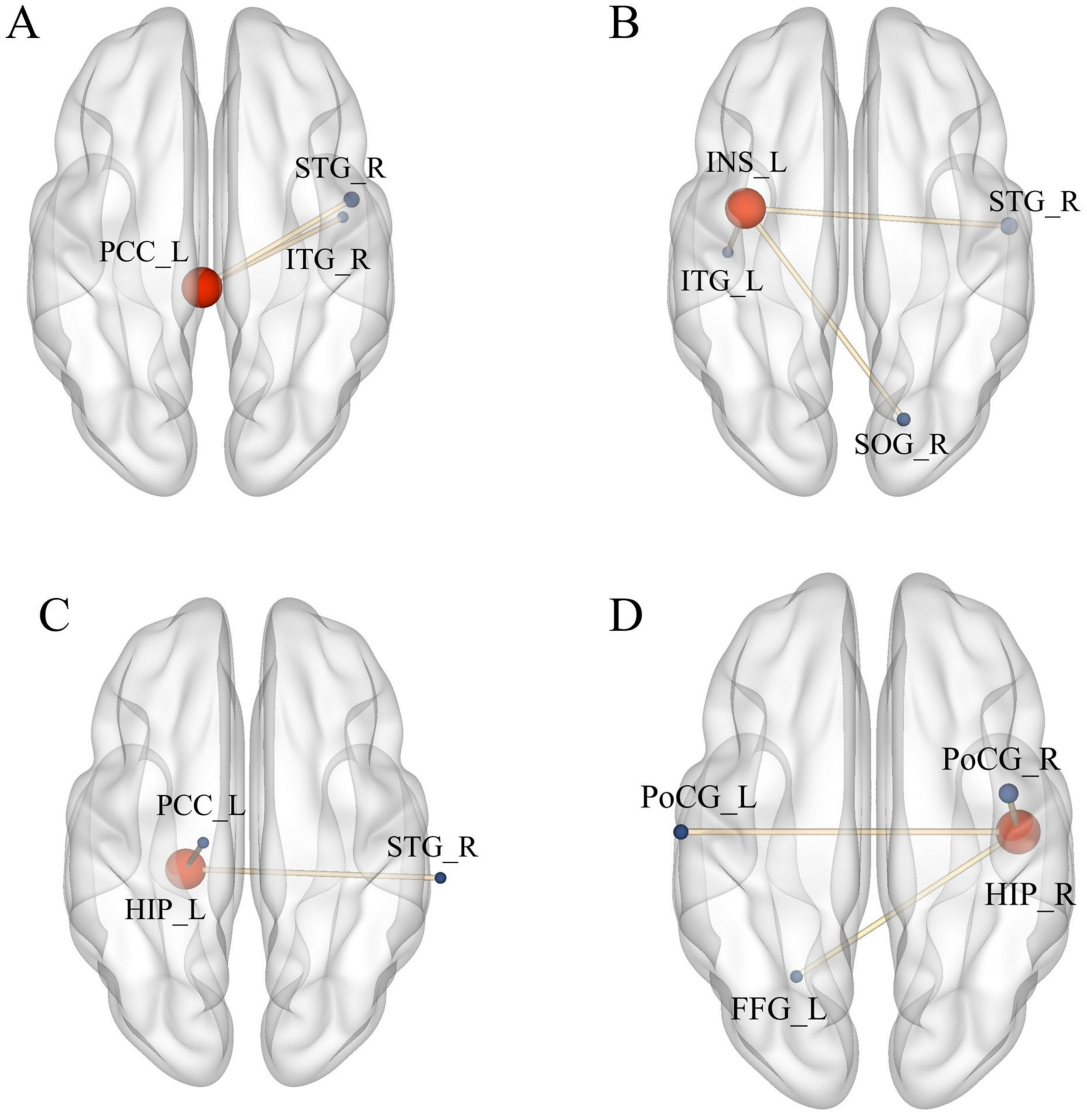
Significant difference in FC between observation group VD patients’ post-treatment and pre-treatment. **(A)** Increased FC between PCC_L and STG_R, ITG_R; **(B)** Increased FC between INS_L, STG_R, SOG_R, and ITG_L; **(C)** Increased FC between HIP_L and STG_R, PCC_L; **(D)** Increased FC between HIP_R and PoCG_L, PoCG_R, FFG_L. Results meet the threshold of voxel *p* < 0.005, cluster *p* < 0.05 (corrected with GRF).

Compared with pre-treatment, FC between left PCC and right superior temporal gyrus (STG), right inferior temporal gyrus (ITG) were increased at post-treatment (Table 4 and Figure 5A); FC between left INS and right superior temporal gyrus (STG), right superior occipital gyrus (SOG), and left inferior temporal gyrus (ITG) were increased at post-treatment (Table 4 and Figure 5B); FC between left HIP and right STG, left PCC were increased at post-treatment (Table 4 and Figure 5C); FC between right HIP and bilateral postcentral gyrus (PoCG), left fusiform gyrus (FFG) were increased at post-treatment (Table 4 and Figure 5D) (voxel *p* < 0.005, cluster *p* < 0.05, corrected with GRF).

**Table 4:** Comparison of FC between observational group’s post-treatment and pre-treatment.

Regions	Side	Voxels	MNI			*T*-value (peak intensity)
			*x*	*y*	*z*	
PCC	L					
STG	R	76	48	0	0	4.781
ITG	R	49	45	−6	−24	3.774
IINS	L					
STG	R	167	54	−9	−12	4.789
SOG	R	137	18	−75	15	4.351
ITG	L	96	−42	−18	−21	5.275
HIP	L					
STG	R	42	66	−30	21	4.213
PCC	L	42	−15	−18	39	4.835
HIP	R					
PoCG	R	189	42	−3	27	7.109
PoCG	L	99	−60	−15	45	4.426
FFG	L	70	−24	−60	−15	5.275

The result was corrected for GRF with voxel *P* < 0.001, cluster *P* < 0.05. MNI, Montreal Neurological Institute. A positive *T*-value indicates an increased FC.

### 3.4 Correlation analysis

**Figure 6:**
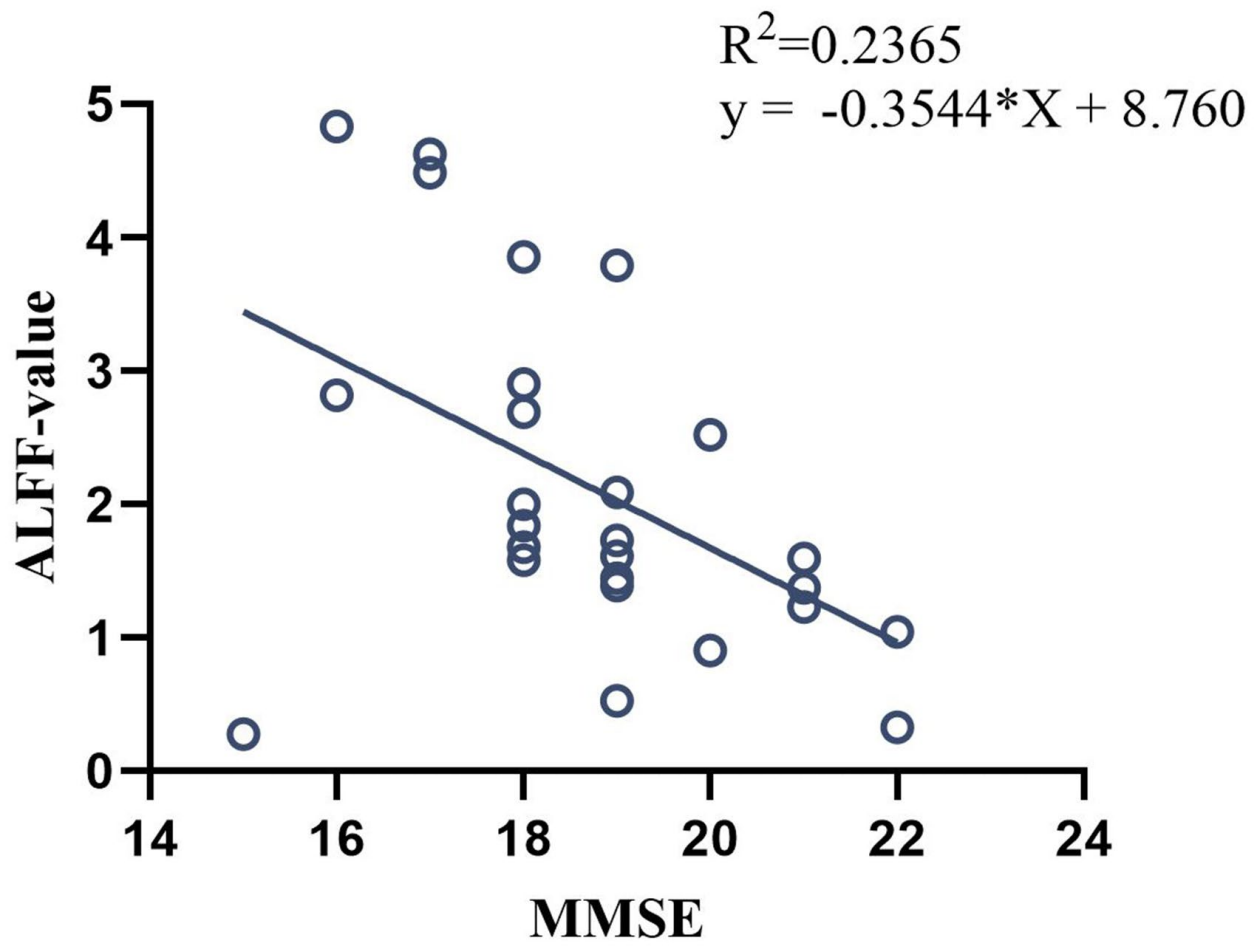
Correlation analysis between ALFF value in pre-treatment VD patients and MMSE scores.

In pre-treatment patients with VD, ALFF value in left MTG was negatively correlated with the MMSE scores (*p* < 0.05, *r* = −0.486, Figure 6), and no significant correlation was found between the rest of the ALFF value and MMSE and MoCA scores.

**Figure 7:**
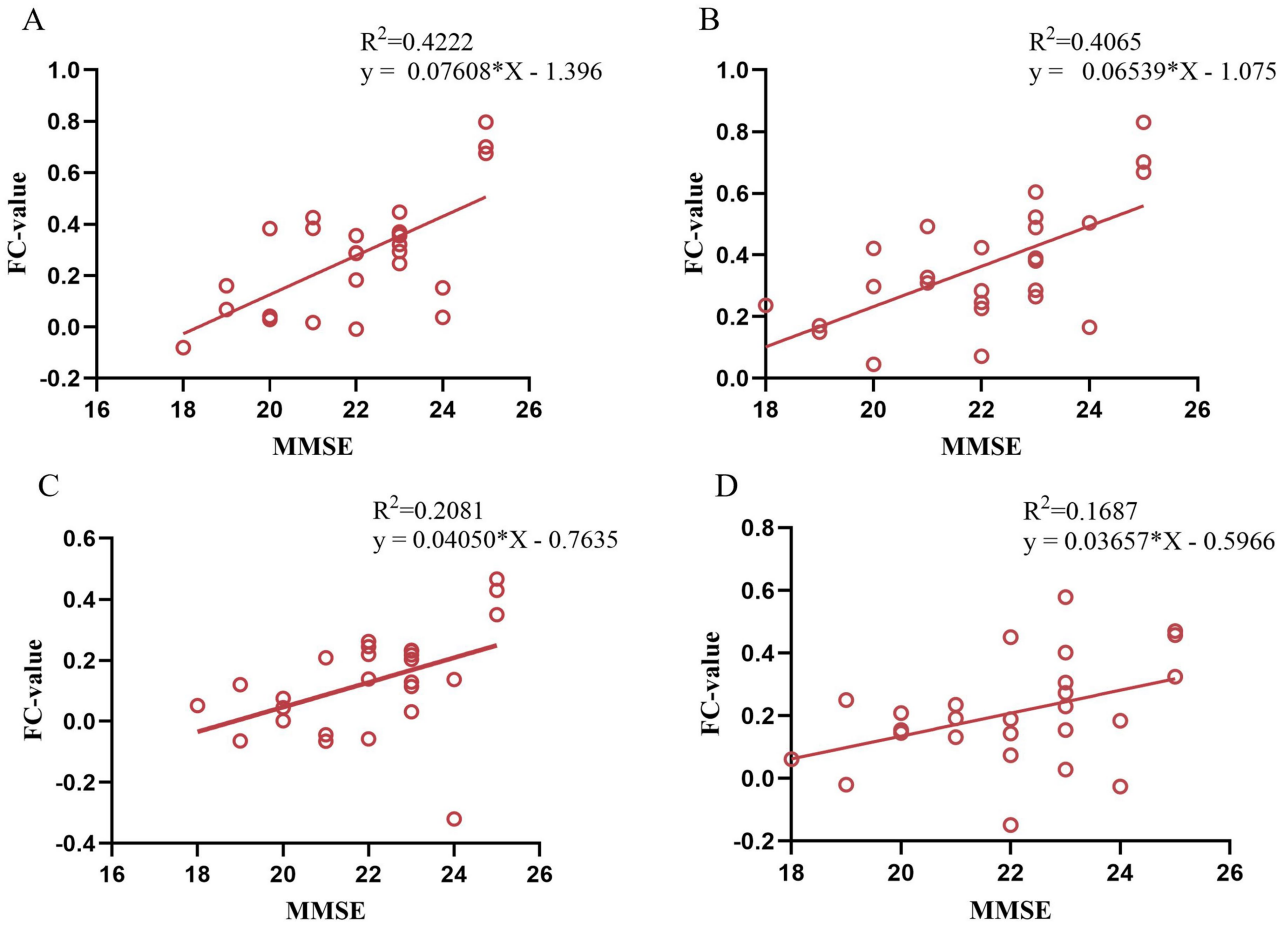
Correlation analysis between FC value in post-treatment VD patients and MMSE scores. **(A)** Correlation between FC (left PCC-right STG) and MMSE scores; **(B)** Correlation between FC (left PCC-right ITG) and MMSE scores; **(C)** Correlation between FC (left HIP-left PCC) and MMSE scores; **(D)** Correlation between FC (right HIP-left FFG) and MMSE scores.

After moxibustion treatment of VD patients, the FC value between left PCC and the right STG/ITG were positively correlated with the MMS scores (*p* < 0.05, *r* = 0.650/*p* < 0.05, *r* = 0.638, Figures 7A,B), the FC value between left HIP and left PCC were positively correlated with the MMSE scores (*p* < 0.05, *r* = 0.456, Figure 7C), and the FC value between right HIP and left FFG was positively correlated with the MMSE scores (*p* < 0.05, *r* = 0.411, Figure 7D).

## 4 Discussion

In this study, moxibustion was used to treat VD, and the results showed that the MoCA and MMSE scores of VD patients increased significantly after treatment, and the clinical efficacy of the patients were significant, and previous clinical studies also shown that acupuncture and moxibustion had a better clinical efficacy in the treatment of VD (8). According to the theory of traditional Chinese medicine, the cognitive function of patients can be improved by stimulating specific acupoints, and the cognitive impairment of patients can be improved. The method of moxibustion is a simple and easily operable technique, GV20 spacer moxibustion with an aconite cake, GV14 and GV24 use hanging moxibustion, combining the warming effect of moxibustion, the pharmacological effect of moxa, as well as the specific effects of these acupuncture points, aiming to achieve disease prevention and treatment. However, there is a lack of information on the effects of moxibustion treatment on brain function in VD patients. Therefore, this study utilized fMRI technology to further investigate the central mechanism underlying the effects of moxibustion in treating VD.

ALFF is an fMRI analysis method that characterize changes in brain function in local brain regions, and its value correlate with the strength of local neural activity in the brain, allowing the study of spontaneous neuronal activity in the brain (22). The results of this study showed that the differential brain regions of ALFF in VD patients compared with healthy control were HIP, PCUN, MTG, PCC, and INS. Among them, the HIP, temporal lobe, PCUN, and PCC are important nodes of default mode network (DMN) (23), which is considered to be a brain network that is closely related to memory, comprehension, and executive functions, and it has been found that the DMN may be one of the important networks contributing to cognitive dysfunction (24), and its nodes may be used to assist in the diagnosis of cognitive impairment (25). Previous studies (26) have found that ALFF values in the DMN (i.e., the PCC/PCUN) of cognitively impaired patients correlate with patients’ MoCA scores, and that patients’ overall cognitive dysfunction may be due to abnormal activity in DMN brain regions. Among them, the MTG is considered to be an important brain region related to semantic and working memory, which is associated with memory and information processing speed (27), and in our study, we found that elevated ALFF values in the MTG of VD patients were negatively correlated with the patients’ MMSE scores, which further suggests that the cognitive decline in VD patients is related to the neuronal activity in the brain regions associated with DMN (i.e., the MTG). INS is located deep in the lateral sulcus of the brain and has a direct structural connection to the motor cortex and the autonomic nervous system, and is a central hub of the brain with an integrative function for a variety of functions, including cognitive and social–emotional processing (28). One study (29) also confirmed the abnormal network function of the INS in patients with cognitive impairment and suggested that alterations in the sub-network of the INS could serve as a potential biomarker for patients with cognitive impairment. In the present study, the ALFF values of INS were reduced, indicating the presence of functional impairment of INS in VD patient, which is consistent with the results of previous studies.

The results of this study suggested that there were abnormal activities in DMN, INS, and other related brain regions in VD patients, so the above brain regions were selected as ROIs to further explore the possible central mechanism of action of moxibustion in treating VD using FC analysis method. Most of the brain regions with FC alterations in VD patients after moxibustion treatment were located in the DMN. FC between left PPC and the right STG and the right ITG were increased, FC between the left HIP and the right STG and left PCC were increased, FC between the right HIP and the bilateral PCC and the left FFG were also increased. The results showed the presence of alterations in the FC of brain regions within the DMN, as well as alterations in the FC of the DMN with other associated brain regions. Numerous studies have shown that HIP is an important brain region for cognitive function (30, 31), and PCC is involved in the encoding process of situational memory, which is closely related to memory function (32, 33), and both are important nodes in DMN. It was found (25) that HIP and PCC are the key brain regions highly affected in DMN in patients with cognitive impairment, which is manifested by decreased FC, and similarly a study observed decreased FC in the PCC in patients with cognitive impairment (34), and another study also found that HIP and temporal lobe FC could be increased after acupuncture treatment (35), and the results of which were generally in agreement with the present study. The results of this study suggested that after moxibustion treatment, the overall FC strength of two key brain regions in DMN (i.e., the PCC/HIP) was increased. The superior occipital gyrus is the main node of the visual network, and previous studies have found that patients with cognitive disorders have altered visual network function, which may be more closely related to spatial recognition cognition (36, 37). The results of this study suggest that after treatment, FC of INS with the STG/ITG and occipital lobe increased in VD patients.

This study further observed that the enhanced FC of PCC and STG/ITG, and the enhanced FC of HIP and PCC/FFG gyrus were positively correlated with the MMSE scores. In patients with VD, cerebral vascular ischemia and hypoxia impair the information transmission pathways among brain regions such as the PCC, HIP, and temporal lobe. This impairment leads to dysfunction in the coordination of memory encoding and semantic processing. In the present study, moxibustion was applied to acupoints GV20, GV14, and GV24 of the Governor Vessel in a standardized manner. It may regulate cerebral blood perfusion, promote neuroplasticity, and enhance the FC strength among core nodes of the DMN. These effects improve the efficiency of memory encoding, consolidation, and retrieval, ultimately leading to significant improvements in patients’ basic cognitive abilities (e.g., memory, orientation, and language comprehension), which is reflected by the marked increase in MMSE scores. Notably, no significant correlation was observed between neuroimaging indicators (ALFF/FC) and MoCA scores. This difference may stem from the differences in the core assessment dimensions of the two types of scales. The MMSE scale focuses on basic cognitive functions, while the MoCA scale, in addition to basic cognitive dimensions, also includes complex cognitive assessment items such as executive function. These results suggest that moxibustion may primarily improve basic cognitive functions in VD patients by targeting the restoration of DMN functional integrity, rather than directly regulating brain networks associated with complex cognitive functions. This also clarifies that the core efficacy characteristic of moxibustion treatment for VD may be its specific advantage in improving basic cognitive function.

## 5 Conclusion

In conclusion, this study clarifies that patients with VD exhibit brain function alterations, with the DMN identified as a key network affected by such abnormalities. Moxibustion effectively improves cognitive dysfunction in VD patients, and its potential mechanism may involve promoting functional recovery of the DMN and facilitating effective functional reorganization. This finding highlights the great potential of moxibustion for the early intervention and treatment of VD.

## 6 Limitations

A key limitation of this study lies in its preliminary, small-scale self-controlled design, adopted due to challenges in recruiting VD patients. Specifically, many patients with cognitive impairment struggle to attend outpatient visits regularly and receive standardized treatment independently, often because of their advanced age and cognitive deficits. Furthermore, the scale evaluation primarily assesses the overall cognitive function of VD patients, without detailed scoring for specific domains such as spatial memory and executive function. This limitation precludes a comprehensive elucidation of the mechanisms underlying the improvement of spatial memory and executive function through moxibustion therapy in VD. Future research protocols will be refined to address this gap. Nevertheless, even with a relatively small sample size, the observed alterations in functional connectivity within the DMN following VD treatment provide preliminary yet valuable insights into the neural mechanisms of moxibustion therapy for vascular dementia.

## Data Availability

The original contributions presented in the study are included in the article/supplementary material, further inquiries can be directed to the corresponding author.
